# Evaluation of Different Cleaning Strategies for Removal of Contaminating DNA Molecules

**DOI:** 10.3390/genes13010162

**Published:** 2022-01-17

**Authors:** Martina Nilsson, Hanne De Maeyer, Marie Allen

**Affiliations:** 1 Forensic Section, Division of Investigation, Stockholm Police Region, Swedish Police Authority, 106 75 Stockholm, Sweden; martina.nilsson@polisen.se; 2 Department of Immunology, Genetics and Pathology, Uppsala University, 751 08 Uppsala, Sweden; hanne.demaeyer@igp.uu.se

**Keywords:** DNA molecules, decontamination, cleaning strategies

## Abstract

Decontamination strategies and their efficiencies are crucial when performing routine forensic analysis, and many factors influence the choice of agent to use. In this study, the effects of ten different cleaning strategies were evaluated to compare their ability to remove contaminating DNA molecules. Cell-free DNA or blood was deposited on three surfaces (plastic, metal, and wood) and decontaminated with various treatments. The quantities of recovered DNA, obtained by swabbing the surfaces after cleaning using the different strategies, was analyzed by real-time PCR. Large differences in the DNA removal efficiencies were observed between different cleaning strategies, as well as between different surfaces. The most efficient cleaning strategies for cell-free DNA were the different sodium hypochlorite solutions and Trigene^®^, for which a maximum of 0.3% DNA was recovered on all three surfaces. For blood, a maximum of 0.8% of the deposited DNA was recovered after using Virkon^®^ for decontamination. The recoveries after using these cleaning strategies correspond to DNA from only a few cells, out of 60 ng of cell-free DNA or thousands of deposited blood cells.

## 1. Introduction

DNA analysis of trace evidence can present many challenges in a forensic investigation, such as samples that contain low quantities of biological material and degraded DNA. However, improved typing kits for forensic analysis may allow STR profiling on DNA extracted from only a few cells [1,2]. In addition, using mitochondrial DNA (mtDNA) instead of nuclear DNA (nDNA) markers allows testing of even lower DNA input amounts, due to the high copy number of mtDNA in each cell [3,4]. Another challenge, especially when analyzing mtDNA, is that exogenous DNA can be present in the samples and cause ambiguous or erroneous results. Contaminating DNA may be introduced to a sample by the first responding officers, rescue workers, or crime scene investigators present at the crime scene, archaeologists during excavations or later by laboratory personnel during DNA analysis [5,6,7]. Contamination may also originate from other samples processed at the same laboratory (cross-contamination) [6,8] or even from disposables and consumables contaminated during manufacturing [9,10].

Several preventive measures should be taken to reduce the risk of contamination in order to assure a high quality of DNA profiling results. Contamination is monitored by establishing elimination databases with DNA profiles from staff [11], periodic wipe tests, and the use of no template control (NTC) reactions [8]. Moreover, personal protective clothing, disposable consumables, aerosol resistant tips, and dedicated instruments are used to prevent the introduction of contaminating DNA into the laboratory environment and casework samples. A laboratory construction with designated pre- and post-PCR rooms equipped with dead-air cabinets and a unidirectional workflow is also important. In addition, several decontamination strategies (e.g., chemical cleaning agents and ultraviolet radiation [UV]) can be used for the decontamination of laboratory surfaces and equipment. While decontamination agents have various DNA damaging mechanisms, UV radiation is destructive because it oxidizes bases to introduce single- and double-strand breaks in the DNA molecules [12,13,14,15,16,17,18].

This study evaluates the decontamination efficiency of ten different cleaning strategies: ethanol, UV radiation, ethanol in combination with UV radiation, fresh and stored household bleach, DAX Ytdesinfektion Plus, Rely+On^TM^ Virkon^®^, Trigene^®^, DNA Remover^®^, and sodium hypochlorite. Most forensic materials have a majority of the DNA present within cells, but it also exists in free form, so-called cell-free DNA. As cells deteriorate with time and in challenging environments, the cell walls become disrupted, resulting in a release of cell content, including the DNA [19,20]. Therefore, both cell-free (extracted DNA) and cell-contained DNA (blood) were placed on three different surfaces (plastic, metal, and wood) in the experiments. The artificially contaminated surfaces were cleaned with the different treatments and then swabbed for residual DNA. Real-time PCR quantification of mtDNA extracted from the collected samples was thereafter used to compare the efficiency of the different cleaning strategies.

## 2. Materials and Methods 

Human male DNA in a 10 μL volume (6 ng/μL, PE Biosystems, Foster City, CA, USA) was deposited on different surfaces. The applied DNA solution consisted of 60 ng DNA, with approximately 18 million mtDNA copies, assuming 2000 mtDNA copies per cell. The mtDNA and nuclear DNA (nDNA) ratio for the DNA sample used in this study was estimated previously (by comparison to a plasmid clone of mtDNA) [21]. As cell-contained DNA, 10 μL of whole blood (unknown DNA concentration) from one anonymous donor was deposited on several surfaces. The sample solutions were deposited in marked 25 mm-wide circles on plastic, metal, or wood surfaces. The wood consisted of a skirting board with one layer of water-based transparent white base paint, the metal of aluminum foil and the plastic of plastic document folders. These surfaces were chosen to mimic common types of material in surfaces of workbenches and equipment in the laboratory environment. The liquid was spread with a pipette tip within the circle and left to dry for two hours.

The different decontaminating strategies to be evaluated were chosen following an informal survey of the most commonly used treatments among European forensic laboratories. The ten strategies selected are described in detail in Supplementary Materials, Table S1. Briefly, the strategies consisted of 70% aqueous ethanol, UV radiation (20 min at a distance of 60 to 70 cm, 254 nm), 70% aqueous ethanol in combination with UV radiation, 15% freshly diluted Klorin Original household bleach (Colgate-Palmolive AB, New York, NY, USA), (3.6% sodium hypochlorite diluted to 0.54%), 15% stored Klorin household bleach (2.7% sodium hypochlorite diluted to 0.4%), DAX Ytdesinfektion Plus, 1% Rely+On^TM^ Virkon^®^, 10% Trigene^®^ Disinfectant Cleaner, DNA Remover^®^, and 0.4% freshly diluted sodium hypochlorite. The commercial cleaning agents were prepared according to the manufacturers’ instructions. The stability of diluted sodium hypochlorite for longer periods has been discussed as the concentration of available chlorine decreases with time [22]. Therefore, both diluted household bleach stored in a dark refrigerator at 8 °C for 80 days and freshly made dilutions were evaluated.

The liquid cleaning agents were administered to the artificially contaminated surfaces by one spray from a calibrated spray bottle. The calibration was performed by weighing five replicates of sprayed volumes followed by adjustments when needed. The cleaned areas were wiped in three circular movements in a similar manner and were performed by the same person using dust-free paper. The cleaned areas were thereafter left to dry for 120 min, with the exception of areas cleaned with Trigene^®^, which was sprayed with a single spray of water before wiping, and then left for 10 min. The entire marked area for each sample was swabbed with a cotton swab (Selefa, OneMed, Danderyd, Sweden) moistened in 0.9% sodium chloride. Five replicates were collected for each test parameter (surface and treatment). No-treatment controls, consisting of the addition of DNA or blood without any decontamination treatment, were collected in three replicates for each surface. The no-treatment controls revealing a maximum DNA yield were used to estimate the ability of the cleaning strategies to remove DNA contamination by comparing the percentage of recovered DNA for the different cleaning strategies. Negative control swabs (background controls) were obtained by sampling three replicates from surfaces without addition of DNA or blood. Thus, at least three replicates were available and analyzed for all different tests.

DNA extraction was performed using the DNeasy Blood and Tissue kit (Qiagen, Hilden, Germany), with the final extracts being eluted in a total volume of 100 μL. Mitochondrial DNA was quantified using a real-time PCR based assay described by Andréasson et al. [21]. This assay was chosen in order to achieve a highly sensitive DNA quantification allowing determination of trace residues of DNA left after cleaning. The PCR reaction consisted of 2X SsoAdvanced™ Universal SYBR Green Supermix (Bio-Rad Laboratories Inc., Hercules, CA, USA), 400 nM each primer (mt-8294F/mt-8436R), and 5 μL DNA extract in a total reaction volume of 25 μL [21]. Real-time PCR was performed using the Bio-Rad CFX96™ Real-Time System (C1000 Touch Thermal Cycler, Bio-Rad Laboratories, Hercules, CA, USA) and Bio-Rad CFX Manager 2.1 software. The PCR conditions consisted of 98 °C for 2 min followed by 40 cycles at 95 °C for 5 s, 60 °C for 20 s, and 95 °C for 5 s. Negative controls were included in all extraction (no swabs added) and quantification (water instead of DNA added) steps.

**Figure 1:**
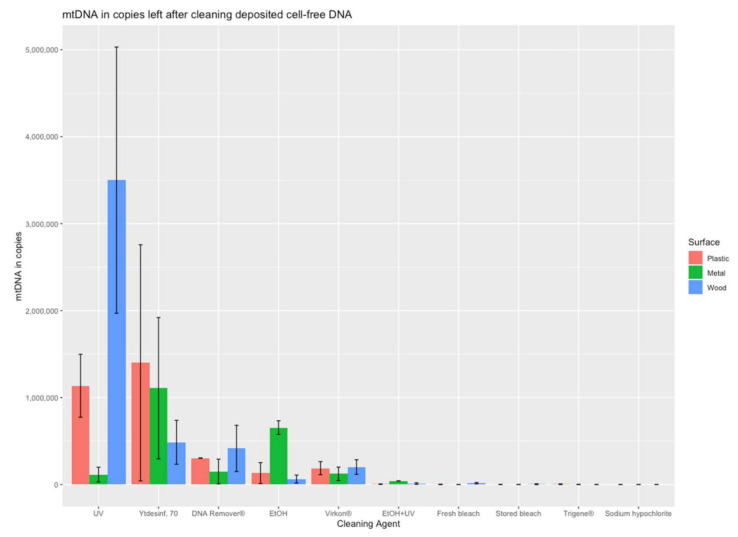
The amount of mtDNA recovered from cell-free DNA deposited on the different surfaces following cleaning with the different strategies. The results are presented as the mean of mtDNA copies (and standard deviations) for a minimum of three replicates recovered from each surface after using the cleaning treatments. The agents are displayed according to the mean recovery for the three surfaces from the highest to the lowest recoveries.

**Figure 2:**
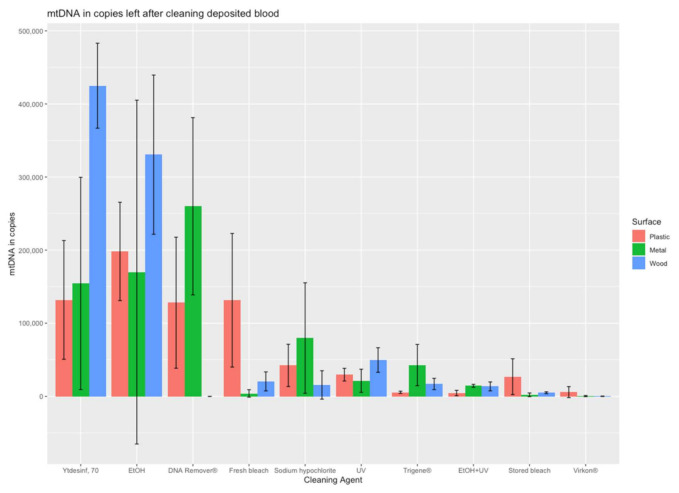
The amount of mtDNA recovered from blood deposited on the different surfaces following cleaning with the different strategies. The results are presented as the mean of mtDNA copies (and standard deviation) for a minimum of three replicates recovered from each surface after using the cleaning treatments. The agents are displayed according to the mean recovery for the three surfaces from the highest to the lowest recoveries.

Data analysis was performed using Microsoft^®^ Excel (version 2019). The 1.5 × interquartile rule was applied to the five biological replicates for each condition to identify potential outliers. Data with and without removal of outliers are presented in Tables S2 and S3. The efficiencies of the cleaning strategies were calculated as a percentage of the positive controls. RStudio^®^ (version 1.3.1093) was used to produce Figure 1 and Figure 2 using the following packages: stats [23], tidyverse, dplyr, ggpubr, ggplot2, and bibtex.

## 3. Results

An evaluation of the decontamination efficiency of ten cleaning strategies was performed by quantifying the amount of mitochondrial DNA (from both cell-free DNA and cell-contained DNA) recovered after the cleaning of contaminated plastic, metal, and wood surfaces. The mtDNA quantification results for each combination of cleaning treatment and surface are illustrated in Figure 1 and Figure 2 and Supplementary Materials, Tables S2 and S3. In general, there were large differences in the efficiency of DNA removal between the tested cleaning strategies.

For the cell-free DNA, recoveries of approximately 52, 32, and 27% of the total 60 ng DNA deposited were observed for the no-treatment controls on the plastic, metal, and wood surfaces, respectively. These quantities were used to compare the recovery after the different cleaning strategies. The background controls generated less than 400 mtDNA copies (out of 18 million copies deposited) and were, therefore, ignored in the further evaluation. The recoveries expressed in mtDNA copies and the percent of recovery relative to the no-treatment controls are shown in Table 1.

The cleaning procedures of ethanol and UV were quite inefficient when used alone, with DNA recoveries of up to 11% and 73%, respectively, from all surfaces. However, the combination of both treatments was much more efficient, with recoveries between 0.1 to 0.7% from the three surfaces. Moreover, fresh bleach, stored bleach, Trigene^®^, and sodium hypochlorite were very efficient in removing DNA, with recoveries from 0.0 to 0.3% from all surfaces. These five decontamination strategies (EtOH+UV, fresh bleach, stored bleach, Trigene^®^, and sodium hypochlorite) resulted in less than 1% of the DNA recovered from the surface. In addition, if accepting up to 5% recovered DNA, Virkon^®^ is also efficient. The least efficient decontamination strategies were UV and DAX Ytdesinfektion Plus, with up to 73% and 19% DNA recovered, respectively.

For cell-contained DNA in the form of blood, the recoveries expressed in mtDNA copies and the percent of recovery relative to the no-treatment controls are shown in Table 2. Also, for blood, the cleaning procedures of UV and ethanol were relatively inefficient when used alone, with recoveries of up to 11% and 70%, respectively, from all surfaces. Again, the combination of both treatments (EtOH+UV) was much more efficient, with recoveries between 0.6 to 2.9% from the three surfaces, representing the second most efficient strategy for blood. The most efficient strategy was Virkon^®^, with recoveries between 0.0 and 0.8% for the three different surfaces. If accepting up to 5% recovered DNA, also stored bleach fulfils the criterion. The least efficient decontamination strategy was DAX Ytdesinfektion Plus, with up to 91% DNA recovered after cleaning.

Large differences were observed between the DNA removal of both DNA sources (cell-free DNA and blood) from the plastic, metal, and wood surfaces, indicating differences in surface absorbance and sampling efficiency. On the wood surface, more than 70% of the added DNA was recovered, and, thus, not efficiently removed by the cleaning with ethanol (for blood), UV (for cell-free DNA,) and DAX Ytdesinfektion Plus (for blood). For these three decontamination strategies, the recoveries differed at least 40% between wood and the other surfaces, indicating that the porous wood presents a challenge for decontamination with large amounts of DNA remaining on the surface (Table 1 and Table 2). For cell-free DNA, UV radiation left the highest amount of mtDNA copies (73% on wood), followed by the second highest, which was DNA in blood left by DAX Ytdesinfektion Plus (27% on metal). DAX Ytdesinfektion Plus left the highest DNA amount from blood (91% on wood) of all agents, followed by aqueous ethanol (70% of blood DNA on wood) and DNA Remover^®^ (43% of blood DNA on metal). It was observed that decontamination of DNA is less efficient in general for DNA from blood compared to cell-free DNA, which may be a result of the protection by the nucleus and the cell wall (Table 1 and Table 2). This is illustrated by the fact that more of the decontamination agents exceed 5% of recovered DNA for blood than for cell-free DNA. Moreover, recovery of more than 5% of DNA from blood was observed on all three surfaces for DAX Ytdesinfektion Plus (ranging from 18 to 91%) and EtOH (ranging from 28 to 70%).

The most successful decontamination strategies identified in this study for cell-free DNA were fresh and stored bleach, Trigene^®^ and sodium hypochlorite. These cleaning strategies resulted in 0.3% or less of cell-free DNA being recovered from all surfaces, which is equivalent to 0.05 ng DNA or approximately 15,000 mtDNA copies (approximately eight cells) out of the 18 million mtDNA copies initially deposited on the surface. In addition, the combination of EtOH and UV was highly efficient on all three surfaces (0.1 to 0.7%). For blood, the best decontamination efficiency was obtained by Virkon^®^ (0.0 to 0.8%). Thus, some of the decontamination treatments were demonstrated as highly efficient in this study, while others were less efficient in removing contamination.

## 4. Discussion

Our results demonstrate that using different cleaning strategies on three different surfaces can remove between 27 and 100% of cell-free DNA and between 9 and 100% of the DNA in the blood. While most treatments showed moderate differences between the recoveries from the three surfaces, the DNA recoveries following UV treatment ranged from 2 to 73% for cell-free DNA and from 4 to 11% for DNA in the blood (Table 1 and Table 2). The differences in the DNA recoveries from the three surfaces may be attributed to the different structures, absorbances, and porousness of the materials as well as the efficiencies of the different treatments. In addition, the agents may result in a chemical reaction with components of the surface, causing for example, corrosion.

In addition to the type of cleaning agents, several other factors can influence the efficiency of removing contaminating DNA during the cleaning process. For example, the mechanical energy from the wiping action, combined with the chemicals used, may enhance the cleaning effect [24]. Moreover, substantial DNA loss is expected in the sampling by swabbing and the extraction process [25]. In this study, the sampling and extraction efficiency was demonstrated by the no-treatment positive controls, resulting in a loss of 48, 68, and 73% of cell-free DNA from the plastic, metal and wood surfaces, respectively. It is important to note, however, that most extraction kits are developed for the purification of cell-contained DNA and not cell-free DNA, which may result in larger losses in this case than for other sample types. The amount of DNA to simulate contamination to be removed or destroyed was very high in this study, with a total of 60 ng of DNA or 10 μL of blood added to the surface. The high amounts of DNA were used to compensate for expected loss in the initial sample preparation process and to challenge the different cleaning strategies efficiently. The added DNA amount is excessively more than expected from most contamination events by aerosols or touched surfaces (without gloves) and may only be possible to transfer to laboratory surfaces by contamination from a concentrated DNA extract or items with relatively fresh blood stains. Despite all factors that may cause variation, this study could demonstrate decontamination efficiency, by measurements of DNA quantities made before and after cleaning.

Apart from efficiency, several parameters are important when choosing a decontamination strategy, such as simplicity of use, safety, and cost. Ethylene oxide is a highly efficient but poisonous agent and, therefore, unsuitable for decontamination in a laboratory environment [26]. Moreover, certain combinations of agents and strategies may be hazardous, such as 1% sodium hypochlorite and 70% ethanol, which form gaseous chlorine and chloroform when combined [17]. It is also essential to allow practical and straightforward daily routines. A commonly used decontaminating agent in laboratories is 1–10% sodium hypochlorite solutions, and a low-cost solution can easily be prepared by diluting household bleach. [17]. A low-cost hypochlorite solution can easily be prepared by diluting household bleach. In this study, the in-house sodium hypochlorite dilutions were made at 15% for easy preparations. Due to discontinuation of the stock solution used for the stored bleach, the bleach solutions were prepared from two different variants of the same brand, Klorin Original and Klorin, with sodium hypochlorite concentrations of 3.6 and 2.7%, respectively. Thus, the 15% dilutions resulted in final Cl_2_ ion concentrations of 0.54 and 0.4% for fresh and stored bleach, respectively. It is, therefore, important to note that the ratio between the fresh and the stored bleach is 1:0.74 rather than 1:1 when comparing the decontamination efficiencies. To further simplify the routines, the lasting properties of diluted household bleach are important. The available chlorine concentration may be affected in hypochlorite solutions when exposed to light, air, and heat, among other factors. This may reduce the stability, but it has been shown that solutions stored up to 200 days at 4 °C are stable [22]. Our study demonstrated that a hypochlorite solution stored at 8 °C for 80 days is not less efficient than the freshly made solution. Therefore, preparing a new solution is not needed more often than every second month if stored properly.

Many cleaning agent solutions contain hazardous chemical compounds listed in their safety data sheets. The treatments used in this study have a variety of health risks. Some are safe (e.g., DNA Remover^®^) with no risks listed, while others present several risks (Supplementary Materials, Table S1). Health risks are very important, but also, the cost is a relevant parameter. For example, DNA Remover^®^ costs 100 times more than household bleach. In contrast, Trigene^®^ has a low cost but is associated with multiple health and environmental hazards. Another important aspect is that the cleaning agent might inhibit the PCR reaction and affect the downstream profiling process. An inhibitory effect has been observed for minute amounts of Trigene^®^ introduced into the PCR reaction, whereas Virkon^®^ or ethanol did not affect STR profiling [14]. All this may influence laboratories final choice of decontamination agent.

Although sodium hypochlorite is highly efficient for the removal of DNA contamination, there are several drawbacks, such as corrosion of metal instruments (e.g., scissors and tweezers). For this reason, alternatives to bleach may be preferred for repeated decontamination of metal items. We performed a limited experiment with metal scissors (stainless steel) submerged in 15% household bleach, DNA Remover^®^, Virkon^®^, and water. Preliminary observations showed that corrosion was apparent after only one day in the bleach solution and after five days for water, while no corrosion was observed after 14 days in DNA Remover^®^ or Virkon^®^ (Table S4).Thus, as Virkon^®^ appears to have a high DNA removal ability, this agent may be a good option when decontaminating metal instruments, despite a somewhat lower ability to remove cell-free contaminating DNA compared to household bleach. However, disposable instruments for evidence sampling and recovery (e.g., single-use scalpels or plastic tweezers) may eliminate the need for a non-corrosive decontamination treatment either completely or at least to some extent. Alternatively, if using sodium hypochlorite for decontamination, rinsing or wiping the surface with distilled water should be performed to avoid corrosion [17].

To conclude, this study showed large differences in the decontamination efficiency between commonly used cleaning strategies on different surfaces and types of traces, cell-free or cell-contained. Of all cleaning agents, six left around 1% or less of either cell-free DNA or DNA in blood on all surfaces. For cell-free DNA, EtOH+UV, fresh bleach, stored bleach, Trigene^®^, and sodium hypochlorite demonstrated the highest efficiency. Out of these, the highest remaining DNA amounts of 0.7% (on metal and with removal of outliers) correspond to 0.14 ng or around 43,000 copies of mtDNA. For cell-contained DNA as blood, Virkon^®^ left less than 1% DNA and the highest remaining amount corresponds to 0.025 ng or around 7500 mtDNA copies. In addition to efficiency, parameters such as health hazards, simplicity, and cost should also be considered. Therefore, different agents or a combination of treatments may be optimal for the decontamination in laboratories performing forensic DNA analysis. Considering our own requirements and priorities, the updated routines for our laboratory will be to perform decontamination with a 15% dilution of stored household bleach for laboratory work surfaces and with Virkon^®^ for metal instrumentation to avoid corrosion.

## Figures and Tables

**Table 1:** The efficiency of cell-free DNA removal after cleaning with different treatments on plastic, metal, and wood surfaces. The table is summarizing the mean in mtDNA copies (and standard deviation) per category (3 to 5 biological replicates). The efficiency (Yield) is expressed as the percentage of recovered mtDNA copies of the no-treatment controls.

Cell-Free DNA/Surface	PLASTIC			METAL			WOOD		
Cleaning Agent	Mean	SD	Yield	Mean	SD	Yield	Mean	SD	Yield
No-treatment controls	9,396,667	4,074,633		5,701,333	4,395,753		4,792,667	3,528,892	
EtOH	131,640	120,771	1.4%	655,333	78,520	11.4%	63,460	45,714	1.3%
UV	1,136,000	361,494	12.1%	115,507	84,110	2.0%	3,500,000	1,530,768	73.0%
EtOH+UV	4724	3020	0.1%	42,567	1570	0.7%	11,480	8569	0.2%
Fresh bleach	1991	2900	0.0%	23	30	0.0%	15,313	8443	0.3%
Stored bleach	918	1238	0.0%	85	77	0.0%	4337	5040	0.1%
DAX Ytdesinfektion Plus	1,399,800	1,357,424	14.9%	1,108,198	812,671	19.2%	486,000	252,391	10.1%
Virkon^®^	188,720	75,615	2.0%	123,350	77,649	2.1%	201,400	84,417	4.2%
DNA Remover^®^	304,333	3055	3.2%	149,910	142,169	2.6%	416,000	265,640	8.7%
Trigene^®^	4223	4026	0.0%	970	1657	0.0%	0	0	0.0%
Sodium hypochlorite	199	151	0.0%	0	0	0.0%	8	16	0.0%
Background controls	15	26		37	65		354	352	

**Table 2:** The efficiency of cell-contained DNA (blood) removal after cleaning with different treatments on plastic, metal, and wood surfaces. The table is summarizing the mean in mtDNA copies (and standard deviation) per category (3 to 5 biological replicates). The efficiency (Yield) is expressed as the percentage of recovered mtDNA copies of the no-treatment controls.

Blood/Surface	PLASTIC			METAL			WOOD		
Cleaning Agent	Mean	SD	Yield	Mean	SD	Yield	Mean	SD	Yield
No-treatment controls	720,000	66,144		607,667	345,048		469,333	150,111	
EtOH	198,200	67,277	27.5%	170,078	235,148	28.0%	330,600	108,933	70.4%
UV	29,640	8644	4.1%	21,308	15,775	3.5%	49,660	16,808	10.6%
EtOH+UV	4530	3678	0.6%	14,475	1987	2.4%	13,624	6104	2.9%
Fresh bleach	131,380	91,357	18.3%	4028	5124	0.7%	20,465	13,006	4.4%
Stored bleach	26,898	24,457	3.7%	2118	2527	0.4%	5003	1221	1.1%
DAX Ytdesinfektion Plus	131,880	81,201	18.3%	154,488	145,119	25.4%	425,000	58,151	90.6%
Virkon^®^	5824	7503	0.8%	395	790	0.1%	160	321	0.0%
DNA Remover^®^	128,060	89,646	17.8%	260,000	121,290	42.8%	0	0	0.0%
Trigene^®^	5598	1517	0.8%	42,775	28,180	7.0%	16,960	7688	3.6%
Sodium hypochlorite	42,310	28,842	5.9%	79,700	75,524	13.1%	15,618	19,392	3.3%
Background controls	15	26		37	65		354	352	

## Data Availability

Not applicable.

## Supplementary Materials

The following are available online at https://www.mdpi.com/article/10.3390/genes13010162/s1: Table S1, Overview of the cleaning strategies; Table S2, Overview of the mtDNA data in copies from cell-free DNA samples; and Table S3, Overview of the mtDNA data in copies from blood samples; Table S4: Cleaning of scissors_HDM.
