# Supplementary material for: Evaluation of Different Cleaning Strategies for Removal of Contaminating DNA Molecules
Source: Genes (Basel). 2022 Jan 17;13(1):162. doi: 10.3390/genes13010162 (PMC8775027; doi:10.3390/genes13010162)
Supplement: Supplementary file 1 [file genes-13-00162-s001.zip › Supplementary_Table S1_211230.pdf]

Table S1. Overview of the cleaning strategies. The product name (current name, if applicable), active compound, manufacturer, dilution used in the study, storage method, application method, price and health risks are listed. The price per liter for each agent is expressed in USD based on the available price on the Swedish market).

Cleaning Agent	Active Compound	Manufacturer	Dilution	Storage	Application	Price per Liter (USD)	Hazards
**Aqueous Ethanol**	99.5% Absolut Finsprit	Kemetyl (Haninge, Sweden)	70% in aqueous solution	Stored in a trigger spray bottle at room temperature	Sprayed once and wiped with a paper towel	$19.37	H225
**UV Radiation**	Philips TUV, UV-C, 30W/G30 T8	Koninklijke Philips N.V. (Amsterdam, The Netherlands)	Not applicable	Not applicable	Radiation for 20 min at 254 nm at a distance of 60–70 cm	Not applicable	Can cause severe injury to skin and eyes
**Aqueous Ethanol and UV Radiation**	99.5% Absolut Finsprit + Philips TUV, UV-C, 30W/G30 T8	Ethanol: Kemetyl (Haninge, Sweden)<br>UV: Koninklijke Philips N.V. (Amsterdam, The Netherlands)	70% in aqueous solution	Stored in a trigger spray bottle at room temperature	Ethanol: sprayed once and wiped with a paper towel<br>UV: radiation for 20 min at 254 nm at a distance of 60–70 cm	Ethanol: $19.37<br>UV: not applicable	Ethanol: H225<br>UV: Can cause severe injury to skin and eye
**Freshly Prepared Household Bleach (Klorin Original)**	3.6% Sodium hypochlorite in water	Colgate-Palmolive AB (Danderyd, Sweden)	15% resulting in 0.54% sodium hypochlorite in aqueous solution	Freshly prepared each day used; Stored in a trigger spray bottle at room temperature	Sprayed once and wiped with paper towel	$0.51	H290, H315, H318, H400, H411
**Stored Household Bleach**	2.7% Sodium hypochlorite in water	Colgate-Palmolive AB (Danderyd, Sweden)	15% resulting in 0.4% sodium hypochlorite in aqueous solution	Prepared 70–80 days before use; stored in a small glass bottle at 8°C in darkness; transferred to a	Sprayed once and wiped with paper towel	$0.51	H290, H315, H318, H400, H411

				trigger spray bottle for further storage			
**DAX Ytdesinfektion Plus**	400 g/kg Propan-2-ol	CCS Hygien A/S (Malmö, Sweden)	Ready-to-use solution	Stored in a trigger spray bottle at room temperature	Sprayed once and wiped with a paper towel	$3.33	H226, H319, H336
**Rely+On™ Virkon®**	Pentapotassium bis(peroxymonosulphate) bis(sulphate) (40–55%)	Antec International Ltd (Sudbury/Suffolk, U.K.)	1% in aqueous solution	Stored in a trigger spray bottle at room temperature	Sprayed once and wiped with a paper towel	$2.64	H315, H318, H412
**Trigene® Disinfectant Cleaner (Distel™ High-Level Medical Surface Disinfectant)**	< 2.3 g/kg didecyl dimethyl ammonium chloride	Tristel Solutions Ltd (Snailwell, U.K.)	10% in aqueous solution	Freshly prepared each day used; Stored in a trigger spray bottle at room temperature	Sprayed once and left for 10 min; sprayed once with water and wiped with a paper towel	$1.40	H302, H314, H315, H317, H318, H400
**DNA Remover® (PCR Clean™)**	Ethoxylated alcohols, C12–14	Minerva Biolabs GmbH (Berlin, Germany)	Ready-to-use solution	Stored in a trigger spray bottle at room temperature	Sprayed once and wiped with a paper towel	$52.18	H302, H318, H400
**Sodium Hypochlorite**	Sodium hypochlorite (3.5% Cl_2) in GPR Rectapur® aqueous solution	VWR International (Radnor, Pennsylvania, U.S.)	0.4% sodium hypochlorite in aqueous solution	Freshly prepared each day used; Stored in a trigger spray bottle at room temperature	Sprayed once and wiped with a paper towel	$2.44	H290, H314, EUH031

Legend H-sentences:

H225: Highly flammable liquid and vapour<br>H290: May be corrosive to metals	H226: Flammable liquid and vapour<br>H302: Harmful if swallowed
H314: Causes severe skin burns and eye damage	H315: Causes skin irritation
H317: May cause an allergic skin reaction	H318: Causes severe eye damage
H319: Causes serious eye irritation	H336: May cause drowsiness or dizziness

H400: Very toxic to aquatic life	H411: Toxic to aquatic life
H412: Harmful to aquatic life	EUH031: Contact with acids liberates toxic gas
